# Topological data analysis for discovery in preclinical spinal cord injury and traumatic brain injury

**DOI:** 10.1038/ncomms9581

**Published:** 2015-10-14

**Authors:** Jessica L. Nielson, Jesse Paquette, Aiwen W. Liu, Cristian F. Guandique, C. Amy Tovar, Tomoo Inoue, Karen-Amanda Irvine, John C. Gensel, Jennifer Kloke, Tanya C. Petrossian, Pek Y. Lum, Gunnar E. Carlsson, Geoffrey T. Manley, Wise Young, Michael S. Beattie, Jacqueline C. Bresnahan, Adam R. Ferguson

**Affiliations:** 1 Department of Neurosurgery, Brain and Spinal Injury Center, University of California, San Francisco, 1001 Potrero Avenue, Building 1, Room 101, San Francisco, California 94143, USA; 2 Tagb.io, 1 Quartz Way, San Francisco, California 94131, USA; 3 Department of Neuroscience, Ohio State University, 460 West 12th Avenue, 670 Biomedical Research Tower, Columbus, Ohio 43210, USA; 4 Department of Neurosurgery, Tohoku University Graduate School of Medicine, Sendai city, Miyagi prefecture 980-0856, Japan; 5 Department of Neurology, San Francisco VA Medical Center, University of California San Francisco, San Francisco, California 94110, USA; 6 Department of Physiology, Spinal Cord and Brain Injury Research Center, Chandler Medical Center, University of Kentucky Lexington, B463 Biomedical & Biological Sciences Research Building, 741 South Limestone Street, Kentucky 40536, USA; 7 Ayasdi Inc., 4400 Bohannon Drive Suite #200, Menlo Park, California 94025, USA; 8 GenePeeks, Inc., 777 Avenue of the Americas, New York, New York 10001, USA; 9 Capella Biosciences, 550 Hamilton Avenue, Palo Alto, California 94301, USA; 10 Department of Mathematics, Stanford University, Building 380, Stanford, California, 94305, USA; 11 Department of Cell Biology and Neuroscience, W.M. Keck Center for Collaborative Neuroscience, Rutgers University, Piscataway, New Jersey 08854, USA; 12 Department of Neurosurgery, San Francisco VA Medical Center, University of California San Francisco, San Francisco, California 94110, USA

## Abstract

Data-driven discovery in complex neurological disorders has potential to extract meaningful syndromic knowledge from large, heterogeneous data sets to enhance potential for precision medicine. Here we describe the application of topological data analysis (TDA) for data-driven discovery in preclinical traumatic brain injury (TBI) and spinal cord injury (SCI) data sets mined from the Visualized Syndromic Information and Outcomes for Neurotrauma-SCI (VISION-SCI) repository. Through direct visualization of inter-related histopathological, functional and health outcomes, TDA detected novel patterns across the syndromic network, uncovering interactions between SCI and co-occurring TBI, as well as detrimental drug effects in unpublished multicentre preclinical drug trial data in SCI. TDA also revealed that perioperative hypertension predicted long-term recovery better than any tested drug after thoracic SCI in rats. TDA-based data-driven discovery has great potential application for decision-support for basic research and clinical problems such as outcome assessment, neurocritical care, treatment planning and rapid, precision-diagnosis.

## Abstract (web summary)

Data-driven discovery in complex neurological disorders has potential to extract meaningful knowledge from large, heterogeneous datasets. Here the authors apply topological data analysis to assess therapeutic effects in preclinical traumatic brain injury and spinal cord injury research studies.

## Graphical abstract

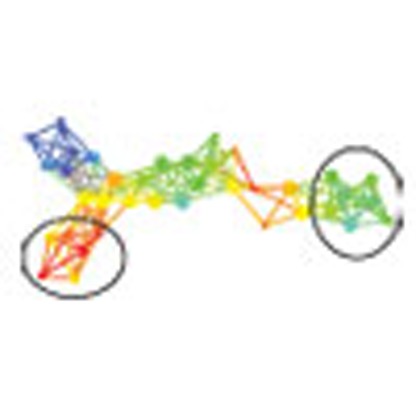

Bioinformatics approaches for precision medicine are gaining momentum as biomedical researchers grapple with overwhelming amounts of data generated by all areas of science in the era of ‘big-data'^1^,^2^. The central nervous system (CNS) injury literature seeks to understand the multifaceted effects of injuries to the brain and spinal cord by collecting high-volumes of detailed information on individual subjects, ranging from histological, physiological and bio-behavioral outcomes to health records from therapeutic trials. The sheer volume of data presents a problem for managing and interpreting therapeutic findings without computational assistance^3^,^4^,^5^. Informatics tools are currently being developed in preclinical and clinical CNS injury studies^6^,^7^, and resources such as the Neuroscience Information Framework (NIF, http://www.neuinfo.org/), ClinicalTrials.gov (www.clinicaltrials.gov) and PubMed (http://www.ncbi.nlm.nih.gov/pubmed/) offer user-friendly query interfaces to bridge knowledge that exists in biomedical research. However, there remains a lack of user-friendly statistical integration and visualization tools that can be applied to primary research data from multifaceted CNS disorders.

In this sense, translation of basic research into clinical therapeutics can be conceptualized as a big-data integration issue. Thousands of studies have been published aiming to characterize spinal cord injury (SCI) and traumatic brain injury (TBI) from a basic scientific view point, yet we still do not fully understand these complicated disorders. In addition, few therapies have navigated successfully through clinical trials into standards for patient care^8^,^9^,^10^. The emerging field of precision medicine seeks to apply analytics and data-visualization tools^3^,^4^ to improve understanding and treatment of complex disorders such as SCI and TBI^11^.

The present study applies a data-analytic approach, topological data analysis (TDA)^12^, for improved discovery of fundamental syndromic injury patterns and assessment of precision therapeutic targeting from preclinical drug trials in SCI and TBI. TDA couples unsupervised pattern detection^13^ and network visualization^12^ to rapidly extract the full syndromic injury disease taxonomy from the full set of inter-correlated biological, behavioral and health outcomes in diverse SCI^14^ and TBI^15^ animal research data. Harnessing a TDA framework, data-driven navigation of the syndromic space is performed by rapid colour-based re-mapping of individual outcomes onto the network to improve interpretation of histopathology, functional recovery and experimental therapeutic effects. TDA helps facilitate the identification of novel relationships in complex, heterogeneous data sets, allowing for data-driven hypothesis generation that may uncover mechanisms for increased morbidity following SCI and TBI. TDA uncovered location-specific impact of SCI and TBI polytrauma on recovery of forelimb function, and differential sensitivity of forelimb measures and locomotion measures in cervical SCI. Application of TDA to preclinical therapeutic trials revealed irreproducible efficacy of methylprednisolone (MP) and minocycline treatment between cervical and thoracic SCI, yet uncovered the novel discovery that perioperative hypertension predicts worse neurological recovery following thoracic SCI.

## Results

**Figure 1:**
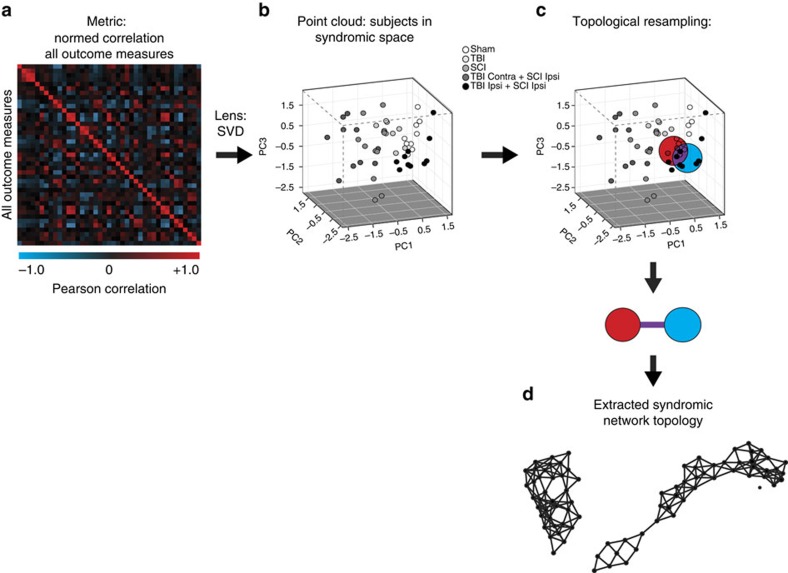
Topological representation of the syndromic space using TDA. (**a**) Data sets containing functional and histological outcomes were analysed using TDA from a bivariate correlation matrix of all outcomes. (**b**) Data were processed with the principal and secondary metric singular value decomposition (SVD) lens to generate the syndrome space. (**c**) TDA resamples the syndromic space many times to link subjects into nodes (red/blue circles) and connects overlapping subjects with edges (purple line) (**d**) to create a robust network topology for rapid visualization and interpretation of outcomes over time.

Initial attempts to visualize the syndromic space following CNS injury in rodents and nonhuman primates have revealed proof-of-concept multivariate relationships of tissue pathology and functional recovery, with each dimension showing specific sensitivity to different injury models^13^,^14^,^16^. Visualizing the syndromic space through traditional methods such as principal components analysis (PCA) requires database querying, statistical coding and graphical programming. These requirements disempower basic researchers and clinicians by limiting rapid and actionable access to syndromic findings (Fig. 1a,b). In contrast, TDA can apply PCA through singular value decomposition (SVD) to reveal the complex multivariate relationship of all predictor and outcome variables simultaneously as a network diagram, where similar individuals are clustered into nodes, and clusters that share one or more individuals are joined by an edge (Fig. 1c). The full syndromic topological map provides a platform for rapid and intuitive exploration of the data set in an unbiased, data-driven manner (Fig. 1d). Once the network is generated, the shape of the data set can be investigated to understand the relationship of each variable across the topological syndromic space to identify groups of clustered individuals that can be further probed for specific relationships among outcomes, validation and targeted hypothesis testing.

### TDA uncovers complex SCI and TBI outcome by injury location

**Figure 2:**
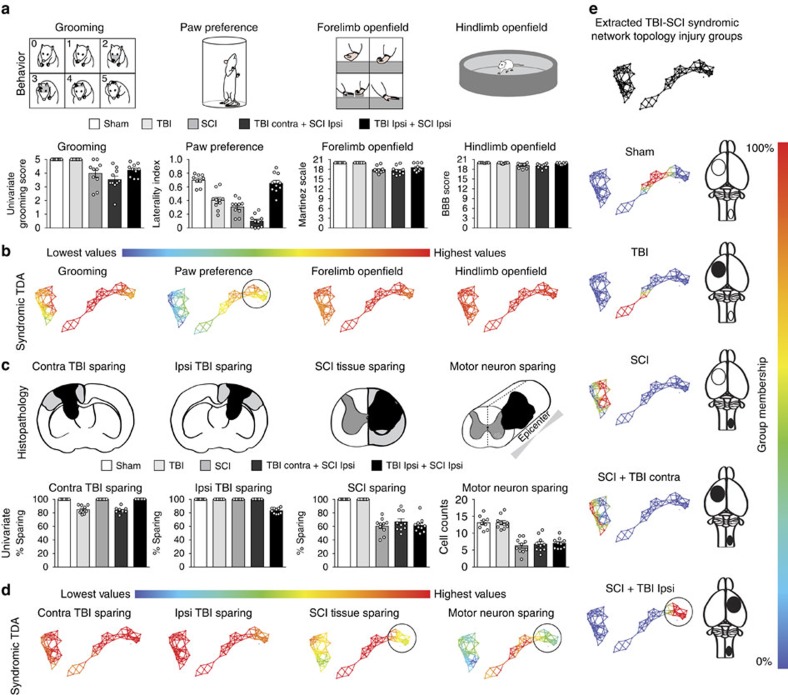
Histo-behavioural network topology of combined TBI-SCI model. (**a,b**) Behavioral outcomes of forelimb function and (**c,d**) histopathology were mapped onto the topological network using TDA. Data from this model shows a distinct recovery pattern depending on whether the combined TBI is contralateral (contra) or ipsilateral (ipsi) to the SCI. (**e**) Each injury group occupies a distinct region of the network topology, highlighted as red nodes for 100% enrichment (heat map) for each particular injury model. Sham controls (*n*=9) and TBI-only (*n*=10) subjects are located in the right cluster. SCI-only (*n*=10) and SCI+TBI contra (*n*=10) are both located in the left cluster. SCI+TBI ipsi (*n*=10) interestingly are grouped next to the sham subjects in the right cluster (circled part of the network), due to a syndromic functional recovery similar to shams (**a**), despite showing no difference in pathology compared with subjects with SCI alone or SCI+TBI contra (**c**). All outcome averages and injury models were exported into an HTML figure (Supplementary Software 1) for rapid visualization and user-guided exploration of the syndromic topological space in this data set.

To test the application of TDA to CNS injury research, we assessed the syndromic network topology of a recently developed rodent model of combined SCI and TBI. The results of the functional and histopathological deficits of this model have been described at the univariate level^15^, with only a subset of endpoints reaching significance (Fig. 2, bar graphs), leading to potentially unclear conclusions about outcome. TDA combined with SVD rapidly re-evaluated the findings across all endpoints simultaneously. Subjects were mapped into the network based on functional (Fig. 2a,b; Supplementary Software 1, dropdown) and histopathological outcomes (Fig. 2c,d; Supplementary Software 1, dropdown), showing a distinct separation of each injury model into sub-networks (Fig. 2e; Supplementary Software 1, dropdown). Sham and TBI-only subjects clustered into distinct regions in the network. SCI-only subjects and SCI+TBI contralateral to each other clustered together into a separate sub-network in the topology, demonstrating worse outcome than the other injury groups (Supplementary Software 1, dropdown). In contrast, subjects with SCI+TBI on the ipsilateral side (Fig. 2, circled) clustered near the sham condition in a sub-network that mapped to better performance on measures of forelimb function (Fig. 2a; Supplementary Software 1, dropdown). This multidimensional difference between ipsilateral and contralateral TBI occurred despite equally-sized lesions (Fig. 2b; Supplementary Software 1, dropdown). Although the univariate effects of lesion location were subtle and varied in their statistical significance across endpoints, (Fig. 2a,c), TDA uncovered a dramatic multidimensional effect when the full ensemble of endpoints was used to render the full syndromic space. Together, these TDA findings reveal the clear separation of syndromic features of compound injuries according to location, providing a proof-of-concept for application of TDA in poly-traumatic CNS injury.

### TDA reveals forelimb outcomes most sensitive to cervical SCI

**Figure 3:**
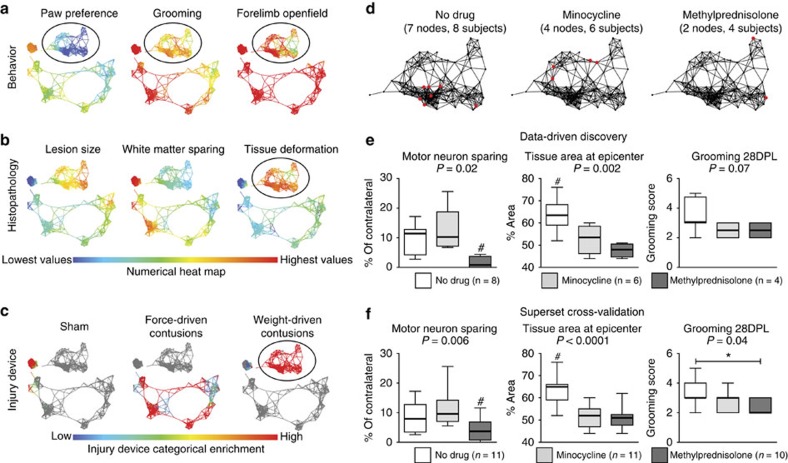
Data-driven discovery of deficits in rats in cervical SCI drug trials. (**a**) Behavioural deficits in forelimb function were identified in the syndromic network (circled area). (**b**) Visual mapping of histopathology patterns in the network did not identify similar patterns to explain behavioral deficits, despite less tissue deformation in this portion of the network. (**c**) Enrichment for injury condition revealed these subjects were given the same type injury (weight-drop contusions, 12.5 mm, Supplementary Software 2). Data-driven exploration of these subjects within the network identified a no-drug controlled trial of minocycline and methylprednisolone (MP). (**d**) Nodes containing subjects significantly enriched for respective drug condition, and 12.5 mm weight-drop injuries were isolated (red nodes) for group comparisons using the Kolmogorov–Smirnov test (KS test). (**e**) The three outcomes with the smallest *P* values from the KS test results were identified in the sub-selection of subjects identified for each treatment condition. Results revealed significant MN loss in subjects receiving MP (*n*=2 nodes, 4 subjects) compared with minocycline (*n*=4 nodes, 6 subjects) and no-drug controls (*n*=7 nodes, 8 subjects) (*P*=0.02, F(2,15)=5.21, *η*^2^=0.41, 1−*β*=0.74), and significantly less tissue area at the injury epicentre in both minocycline and MP-treated subjects, compared with no-drug controls (*P*=0.002, F(2,15)=10.02, *η*^2^=0.57, 1−*β*=0.96). Non-significant functional deficits in grooming were observed 28 days post lesion (DPL) (*P*=0.07, F(2,15)=3.18, *η*^2^=0.30, 1−*β*=0.52). (**f**) Validation of these significant detrimental treatment effects were found in the entire superset of subjects for both MN sparing (*P*=0.006, F(2,29)=6.08, *η*^2^=0.30, 1−*β*=0.85) and total tissue area at epicentre (*P*<0.0001, F(2,29)=19.94, *η*^2^=0.60, 1−*β*=1.0), and grooming at 28 DPL was also significant (*P*=0.04, F(2,29)=3.68, *η*^2^=0.20, 1−*β*=0.63). Box and whisker plots show mean and minimum/maximum range of values. *P* values represent overall treatment effect using one-way ANOVA. *Post hoc* pairwise comparisons between each drug condition identified significant decreases in MN sparing in MP-treated subjects, and more tissue area in no-drug controls (‘#',significantly different from both groups; *P<0.05). All outcomes at each time point, location of injury conditions and treatment groups are mapped onto the HTML network Supplementary Software 2.

To test the application of TDA combined with SVD to SCI, we assembled raw data from several common SCI models, including hemisections, weight-drop and force-driven hemi-contusion injuries to the cervical spinal cord (Fig. 3; Supplementary Software 2, dropdown). Grooming behaviour and paw preference in a cylinder reveal graded levels of recovery (Fig. 3a), that map to lesion size, tissue sparing and deformation (Fig. 3b; Supplementary Software 2, dropdown). However, measures of open-field locomotion for both forelimb (Fig. 3a; Supplementary Software 2, dropdown) and hindlimb (Supplementary Software 2, dropdown) do not show much variability in recovery of function. In the syndromic topology, grooming function has the strongest visual mapping to lesion size (Fig. 3b; Supplementary Software 2, dropdown), whereas recovery of paw preference in the cylinder shows a stronger visual mapping with white matter sparing. Little to no variability is seen in the hindlimb open field (Supplementary Software 2, dropdown), most likely because it was designed to measure hindlimb coordination following bilateral thoracic injuries^17^, whereas these subjects received various grades of unilateral cervical injuries. There was some variance in the measure of forelimb open field for the most severe injuries (Fig. 3a, circle), however, it did not map to the full range of lesion pathology. TDA enabled rapid multivariate visualization of group differences with a quicker turn-around for interpretation.

### Differential mapping of injuries in the syndromic network

To understand how injury models map onto the SCI syndromic space, we recoloured the network using categorical experimental SCI groups (Fig. 3c; Supplementary Software 2, dropdown: sham, hemisection, contusion and so on), and observed biomechanical tissue deformation (μm) measured at the time of injury by servo-feedback position detectors on the SCI contusion devices (Fig. 3b; Supplementary Software 2, dropdown). Each injury group occupies a distinct section of the network (Supplementary Software 2, sham, hemisection, 75 kdyn and 100 kdyn force-driven contusions^18^; 6.25 mm and 12.5 mm weight-drop contusions^19^; red nodes), validating TDA syndromic comparisons of pathology and function across multiple injury models. Mapping tissue changes onto the network (Fig. 3b; Supplementary Software 2, dropdown) confirmed that tissue changes vary as a function of injury group (Fig. 3c; Supplementary Software 2, dropdown) and predict subject positions within the full syndromic network space (Supplementary Software 2). Lesion size shows less variability between the different injury models, with the exception of the most severe 12.5 mm contusions (Supplementary Software 2, dropdown). These larger lesions are confirmed visually in the network, where larger lesion pathology corresponds to 12.5 mm weight-drop injuries and 100 kdyn force-driven injuries. White matter sparing (Fig. 3b; Supplementary Software 2) shows a wide range of graded severities for the contusion injuries (weight drop and force driven), and hemisection injuries show a substantial loss in white matter (Supplementary Software 2, dropdown). This pattern is confirmed in the network, with the distribution of nodes with the most white matter sparing appearing on the perimeter of the bottom flare. Motor neuron (MN) sparing along the rostro-caudal axis of the lesion (Supplementary Software 2, dropdown) is the histological feature most sensitive to injury in this data set; nearly all of the contusion subjects had large-scale loss of MNs, even with the mildest of injuries. This sensitivity of MN loss is visually reflected in the network topology where only the shams and hemisection regions of the flares show MN sparing (Supplementary Software 2, dropdown), illustrating the vulnerability of this cell type to contusive spinal cord damage.

### Visually guided data exploration uncovers drug effects

We identified the nodes within the topology that stood out as having poor functional recovery on grooming and forelimb open field (Fig. 3a, circles), despite nodes in this region showing less-severe injuries based on the degree biomechanical tissue deformation (Fig. 3b, circle). This sub-network was also significantly enriched for 12.5 mm weight-drop contusions, yet we noticed that not all injuries of this type performed so poorly. To probe factors that might contribute to abnormally bad function, we drilled into this effect. We compared subjects in these nodes with 12.5 mm contusions that performed well on forelimb open field using a ranked Kolmogorov–Smirnov (KS) test. The ranked KS test is analogous to a gene-set enrichment analysis here applied to identify predictor and outcome metric sets (rather than gene sets) that are most sensitive to group conditions (hypothesis testing)^20^. KS tests between nodes within the high and low functioning groups uncovered an external predictor (not included in the generation of the network) that could account for functional differences: subjects were part of a preclinical trial of two anti-inflammatory drugs: minocycline and MP and no-drug controls. The network was then recoloured based on treatment condition to highlight nodes enriched for drugs and 12.5 mm weight-drop contusions (Fig. 3d, red nodes, ‘no-drug' control (*n*=11 original subjects; pure nodes=7, *n*=8), minocycline (*n*=11 original subjects; pure nodes=4, *n*=6) and MP (*n*=10 original subjects; pure nodes=2, *n*=4)). KS test results comparing treatment groups enriched in the network suggested significant differences on several outcomes based on *t*-test and KS test *P* values between groups (*P*<0.05; Fig. 3e) in the TDA-identified ‘responder' subjects. To independently confirm this, we performed a one-way analysis of variance (ANOVA) on the TDA-identified subject subsets, confirming significant drug effects on MN sparing (*P*=0.02) and tissue area at the epicentre (*P*=0.002), with other outcomes approaching significance, including grooming at 28 days post lesion (DPL) (*P*=0.07). The effect size (*η*^2^) and power calculation (1−*β*) values (detailed in Fig. 3e legend) suggest that the TDA-identified subset of subjects and outcome metrics for each group had ‘large' effect sizes^21^, yielding high power, despite the limited *n* in the subpopulations of interest. *Post hoc* means testing was performed on significant main effects of treatment using pairwise comparisons between all treatment conditions with multiple-comparison correction. MP-treated subjects had significantly less MN sparing compared with both no-drug controls (*P*=0.03) and minocycline-treated subjects (*P*=0.006), but no difference in MN sparing was found between no-drug and minocycline treatment groups (*P*=0.33). For total tissue area at epicentre, control subjects showed significantly greater tissue compared with both MP (*P*=0.001) and minocycline (*P*=0.006) subjects, but no difference in tissue area was found between MP and minocycline (*P*=0.24). These statistical results suggest that MP significantly reduced MN sparing, and both MP and minocycline impacted total tissue area at the epicentre. After interviewing the original data donors, we discovered that data from this drug trial was not previously published because treatments were thought not to show functional benefits (the ‘file-drawer phenomenon').

The TDA-identified subpopulation analysis suggested that minocycline and MP had effects on a subset of endpoints, in a subset of the individuals. To confirm the generality of these effects, we next tested for the effects of minocycline and MP on MN sparing, total tissue area and grooming function on the full data set (‘superset cross-validation') from this drug trial (Fig. 3f). One-way ANOVA and *post hoc* testing of individual treatment groups was performed with the same criteria as the comparisons in Fig. 3e. Results confirmed a significant main treatment effect for MN sparing (*P*=0.006), with the MP group showing significantly less MN sparing than either minocycline (*P*=0.002) or no-drug controls (*P*=0.04), but not between no-drug and minocycline (*P*=0.21). Total tissue area at the epicentre also had a main treatment effect (*P*<0.0001), with no-drug controls showing significantly more total tissue compared with both minocycline (*P*<0.0001) and MP (*P*<0.0001) groups, but not between minocycline and MP (*P*=0.79) groups. Last, grooming function at 28 DPL had a significant main treatment effect (*P*=0.04), with the MP group showing significant functional deficits compared with no-drug controls only (*P*=0.02), with non-significant grooming deficits found in minocycline compared with no-drug controls (*P*=0.06) or MP (*P*=0.49). Together this suggests that MN sparing and tissue area at the epicentre were the major drivers of the network-detected effects, with more modest contributions by other variables.

### Conflicting cross-validation and irreproducible drug effects

**Figure 4:**
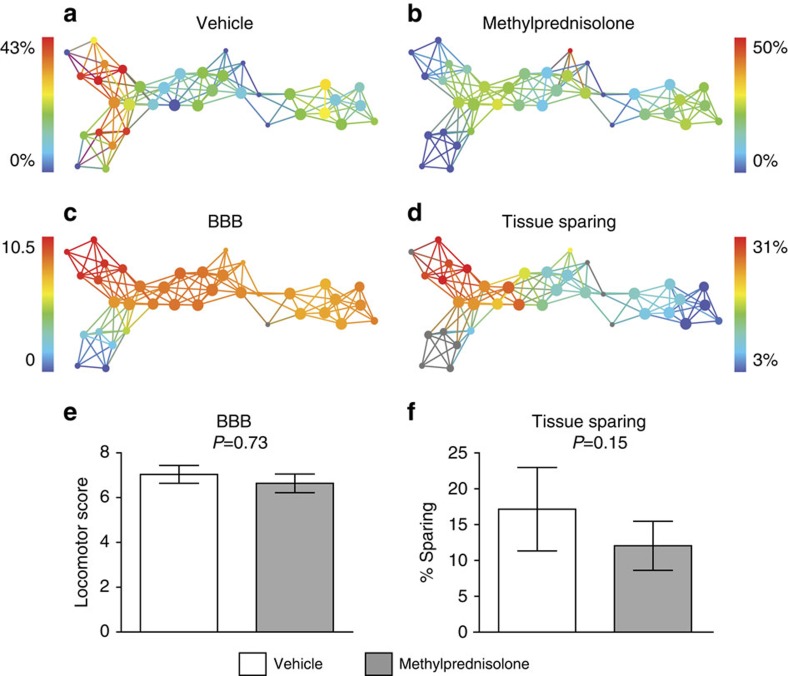
Cross-validation attempt of MP in thoracic SCI L-infinity centrality network. TDA was performed on data mined from the VISION-SCI repository, queried based on subjects that were part of treatment trials testing MP (MP1 and MCP) following SCI (*N*=72). Location of treatment groups within the network for either (**a**) vehicle-treated control or (**b**) MP-treated subjects are shown, however, no nodes were 100% pure for either treatment condition, suggesting treatment was not a significant predictor of placement of subjects within this network. (**c**) BBB recovery and (**d**) total tissue sparing at the injury epicentre were mapped into the network to identify the range of recovery in this data set (red=better recovery, blue=worse recovery). Grouping subjects in the data set based on treatment condition did not reveal the same significant deficits observed in the cervical trial for MP for either (**e**) recovery of locomotor recovery measured by the BBB (*P*=0.73), or (**f**) the total tissue sparing at the epicentre (*P*=0.15). However, there was a trend towards less tissue sparing in subjects that received MP, similar to histopathology observed in cervical SCI (Fig. 3). The most striking difference in the network were subjects who had very large differences in tissue sparing along the top arm of the network, yet showed similar ranges of BBB functional recovery, which are explored further in Fig. 5. Histograms plotted as mean±s.e. Student *t*-test used for significance testing between treatment groups.

TDA-based data-driven discovery revealed a hidden finding in legacy data that MP was potentially detrimental in cervical SCI. To test whether the same might be true in thoracic SCI, we pooled data from the VISION-SCI database^14^ containing other subjects that were part of controlled MP drug trials. A previously conducted trial from the Multicenter Animal Spinal Cord Injury Study (MASCIS)^19^ was identified, which contained a larger cohort of subjects (1996, *N*=72). TDA was performed using the same PCA/SVD lens and norm correlation metric used on the cervical data set (Fig. 3; Supplementary Software 2, dropdown) to cross-validate the detrimental effects of MP on this independent thoracic data set. Identification of nodes in the thoracic network receiving either vehicle control or different doses of MP (coded as MP1, MCP in Supplementary Software 3, dropdown) did not show the same detrimental effects in either functional recovery measured by locomotion with the BBB or tissue sparing at the injury epicentre (Supplementary Software 3, dropdown). A separate analysis on the same data set using TDA was performed with the L-infinity centrality lens, which attempts to cluster subjects in the network based on maximal distance between subjects and how far they are from the group norm^12^. TDA revealed that subjects were distributed along three main flares in the network. Identification of nodes enriched for either vehicle (Fig. 4a) or MP-treated subjects (Fig. 4b) revealed that a maximum of 50% group membership was represented in the red nodes in the network. Location of these nodes for each treatment condition within the network was visually mapped to functional recovery of BBB (Fig. 4c) and tissue sparing at the injury epicentre (Fig. 4d). Querying all subjects within this trial that received either vehicle control (*N*=10) or MP (*N*=12) did not show significant group difference on either BBB locomotor recovery (Fig. 4e, *P*=0.73) or tissue sparing at the epicentre (Fig. 4f, *P*=0.15). The results suggest that MP treatment had no significant effect in the thoracic SCI, in contrast to the deleterious effect observed in the cervical SCI trial (Fig. 3; Supplementary Software 2, dropdown). On the whole, these previously unpublished preclinical findings seem to confirm the lack of definitive data from preclinical trials of MP in SCI.

### Data-driven discovery that hypertension predicts dysfunction

**Figure 5:**
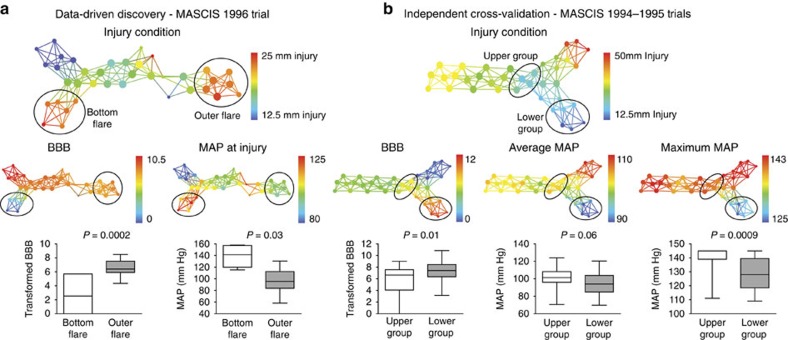
Perioperative hypertension predicts worse recovery after thoracic SCI. (**a**) Exploration of the TDA network from the MASCIS OSU 1996 methylprednisolone trial (*N*=72) revealed a cluster of subjects in the network given the same targeted injury (circled bottom and outer flares) that showed very significant differences in BBB function (*P*=0.0002). A query of variables with significant differences based on KS test results between these two groups uncovered subjects with significant hypertension during SCI surgery (*P*=0.03) clustering in the groups with poorer functional recovery. (**b**) Cross-validation of these relationships between perioperative blood pressure and functional recovery was performed in a separate group of test subjects from the same 3-year drug trial (MASCIS 1994–1995, *N*=154) with matching outcome measures and subject grouping. Visually guided identification of subjects in the network given the same injury condition (circled upper and lower groups) but showing poorer functional recovery on the BBB scale (*P*=0.01) uncovered the same significant detrimental effect of hypertension during SCI surgery on recovery (*P*=0.06), specifically when assessing peak MAP values recorded during surgery (*P*=0.0009). Box and whisker plots show mean and minimum/maximum range of values. *P* values obtained using student *t*-test for significant differences between groups.

Application of TDA in the context of cross-validation testing of MP treatment following thoracic SCI revealed an unexpected and much stronger predictor of neurological recovery than any of the drug conditions. Visually guided exploration of TDA sub-networks uncovered unusually large differences in functional recovery on the BBB locomotor scale in putatively identical injury severities. We isolated these disparate subject populations and categorized them into groups for further comparisons (Fig. 5a, circles). Nodes containing subjects that received identical 25 mm weight-drop contusions were grouped and compared with a KS test to identify measures that significantly differed between these two groups and also mapped to significant functional differences on the BBB (*P*=0.0002). This data-drill down revealed that blood pressure spikes at the time of SCI significantly differed between high and low locomotor recovery subgroups (KS=0.7, *P*=0.03), suggesting that subjects with poorer outcome may have had hypertensive mean arterial pressure (MAP) at the time of injury.

### Cross-validation and confirmation of hypertension hypothesis

The data-driven discovery of hypertension as a major predictor of SCI recovery from semi-structured big-data could potentially represent a ‘capitalization on chance'^22^. To explicitly rule this out, we performed two waves of additional analyses. First, we independently cross-validated the TDA-based data-driven discovery, by curating an additional data set from subjects with less-severe injuries (12.5 mm) from a separate round of the MASCIS trial (1994–1995, *N*=154) (Fig. 5b) queried from the VISION-SCI repository. Nodes within the network that received thoracic 12.5 mm weight-drop injuries showed distinct subpopulations with significant differences in BBB locomotor recovery (Fig. 5b, circles, *P*=0.01). KS testing of the good versus bad recovery subgroups within this network confirmed that hypertensive events (maximum MAP) during surgery predicted lower locomotor recovery in the chronic phase (KS=0.6, *P*=0.0009).

Second, we explicitly tested the formal hypothesis that perioperative hypertension predicts long-term outcome using a repeated measures general linear model (GLM) on the 1996 and the 1994–1995 data sets. Explicit hypothesis testing separately confirmed the hypothesis that perioperative MAP (covariate) predicted poorer functional recovery of BBB (dependent) between 1 and 6 weeks post injury (repeated measure). In both data sets, post-injury MAP (15 min after SCI) significantly predicted the main effect of recovery of BBB locomotion following injury (1996, F(5,20)=3.701, *P*=0.02; 1994–1995, F(5,110)=2.671, *P*=0.03).

### TDA-based data-driven discovery versus traditional tools

**Figure 6:**
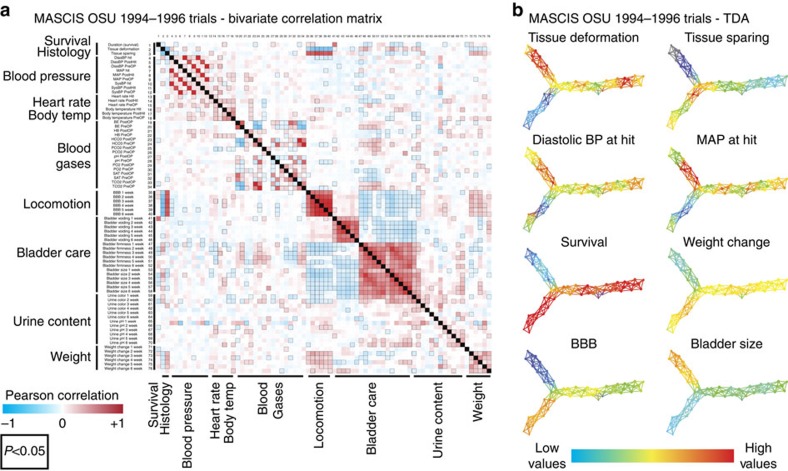
Comparing traditional tools to TDA in MASCIS data set. (**a**) A bivariate correlation matrix was generated for every outcome measured over time along with measures of heart rate, blood pressure and blood gases before, during and after surgery. Each variable is correlated to every other variable, with clusters of similar measures represented with larger text, with specifics about each measure and collected as the same times post injury (1–6 weeks post injury). Histology includes tissue deformation and tissue sparing. Blood pressure includes diastolic pressure, mean arterial pressure and systolic pressure at time of hit, 15 min after hit (PostHit) and 15 min before hit (PreOP). Similar time bins were collected for heart rate and body temperature. Blood gases were measured only either before (PreOP) or after (PostOP) injury. Locomotion was measured between 1 and 6 weeks using the BBB scale. Bladder function was monitored daily and binned across each week post injury for bladder voiding/expression, firmness, size and urine content was recorded for colour and pH. Weight change between each week post injury was also recorded to assess health. Numbers along the *y*-axis are reflected in the *x*-axis to line up variable comparisons. The heat map represents either negative (blue) or positive correlations between each variable within the matrix, with significant correlations (*P*<0.05) highlighted with black boxes. Although this method of visualizing correlations is useful for understanding how different measures all relate to each other within the context of all other comparisons, it does not allow for mapping of each test subjects placement within the network based on all these complex relationships. (**b**) TDA of the same data set reveals the distribution of every subject within the network, from all subjects in the entire OSU MASCIS trial (1994–1996, *N*=334). TDA revealed the same visually guided relationships between perioperative blood pressure and autonomic and locomotor dysfunction following SCI identified in Fig. 5. Complete mapping of all outcomes and perioperative measures of vitals and blood gases over time were exported into an HTML viewer (Supplementary Software 4).

The fact that TDA-guided discovery uncovered a novel finding that was hiding in plain sight in 20-year-old data, provides strong potential support for this approach. However, we wondered whether a similar set of results could have been revealed using traditional analytics. To test this, we pooled all data from MASCIS (*N*=334) in the VISION-SCI repository and performed side-by-side bivariate correlational analysis and TDA (Fig. 6). Pearson correlation confirmation of the significant inverse correlation between elevated perioperative blood pressure and BBB functional recovery was performed by plotting a bivariate correlation matrix for MASCIS OSU trial subjects (*N*=334) for all measures of survival, histology, perioperative vitals and blood gases, functional recovery, bladder health and weight over 1–6 weeks post SCI (Fig. 6a). Blood pressure measures showing the most significant inverse correlations to BBB recovery were confirmed, with elevated diastolic blood pressure at the time of injury, showing the most significant negative correlations at multiple time points (3–6 weeks), with MAP and systolic blood pressure only showing a significant correlation to BBB deficits at 5 weeks post injury. Additional measures also showed significant correlations to BBB recovery in this large correlation matrix, including expected ones such as tissue pathology, bladder care complications and weight gain, with additional measures of body temperature and several blood gas measures during surgery (bicarbonate; blood pH; total carbon dioxide; and partial pressure of carbon dioxide) also showing significant correlations to BBB. Taken together, we found that the major changes in MAP predicting long-term motor impairment, typically occurring in the range between 100 and 143 mm Hg for upwards of 5 min at a time, occurred immediately post injury, during surgery and in the recovery phase in the animal neuroICU.

Although visualization and interpretation of the complex interactions between all the variables in this data set can, in theory, be achieved by simple correlation, this approach does not identify clusters of subjects that are most sensitive to these interactions across the full spectrum of variables. Navigating the same data set with TDA creates a syndromic map of all subjects based on the full network of correlations, enabling rapid comparative hypothesis testing about factors such as injury condition, recovery rate, autonomic factors or even gender differences (Fig. 6b, Supplementary Software 4, dropdown). All outcomes measured over time, including BBB locomotion, bladder function, weight, and perioperative blood pressure and blood gases were mapped onto the network for each time point (Supplementary Software 4, dropdown). Additional mapping of enrichment for gender differences in the network revealed that subjects within the nodes that showed the strongest relationship between perioperative hypertension and BBB recovery were mostly males. Due to bladder complications being more pronounced in males following SCI^14^, and the strong correlation between bladder function and health and recovery of locomotion, males may be more sensitive to the complications of hypertension during surgery, potentially contributing to more autonomic complications post injury, leading to increased morbidity. This TDA-based hypothesis discovery has served to accelerate ongoing interests in our centre in linked preclinical experimental and prospective clinical observational trials of critical care variables including MAP and the use of pressors^23^,^24^ to assess the impact of hypertension, as well as hypotension on recovery of function after SCI.

## Discussion

We report the novel application of TDA to extract the fundamental shape of the multidimensional syndromic space after CNS damage, using preclinical TBI and SCI data as illustrating examples. TDA-based data-driven analyses revealed a set of important findings, some of which dramatically confirmed existing ‘hunches' in the published literature, whereas others represented novel findings that were not previously identified, even in legacy data sets (for example, 20-year-old MASCIS studies). As an illustration of value for confirmatory analysis, TDA revealed a dramatic interaction between SCI and concurrent TBI that depended on anatomical location of brain lesions. Although this effect has previously been reported, it only reached significance on a subset of univariate endpoints^15^, whereas TDA revealed this effect to be very large and robust at the network level. As an illustration of value for exploratory hypothesis generation, TDA identified potential detrimental consequences of MP treatment on tissue pathology in cervical SCI, and to a lesser extent in thoracic SCI. In attempting to cross-validate this observation, we discovered a novel previously unrecognized relationship between perioperative hypertension and poorer long-term functional recovery. The relationship between perioperative hypotension (<85 mm Hg MAP) and poorer neurological recovery was recently reported in humans with SCI^23^, providing a basis for clinical relevance in early neurocritical care on outcomes. However the relationship between acute hypertension has not been reported. Further investigation in humans with SCI needs to be conducted to determine if the findings presented in the current study translate into humans. While the practice guidelines for treatment of acute SCI include avoidance of hypotension, there is little experimental data to support this, and the role of hypertension in outcomes has received less attention^23^,^25^,^26^. The findings presented here suggest that both extremes in MAP may contribute to increased morbidity following injury. Additional prospective experiments are currently underway in rats to test the mechanism by which hypertension exacerbates functional deficits following SCI. Based on the literature, the leading mechanistic candidates include increased oedema cord^27^, increased hemorrhage^28^, blood–brain-barrier breakdown and influx of inflammatory cells and cytokines around the injury. Such effects may promote a more cytotoxic spinal cord micro-environment contributing to increased morbidity^25^,^29^,^30^,^31^,^32^,^33^,^34^,^35^. Taken together with the increased autonomic complications that exist in patients following SCI^36^ (for example, autonomic dysreflexia), which may be triggered by haemodynamic events, hypertension during acute management of SCI may pose a significant risk for patients. This suggests that careful monitoring of blood pressure in acute patients may need to be considered for both the upper (hypertension) and lower limits (hypotension), as both extremes may impact neurological recovery.

The present application of a big-data-analytic tool for novel discovery has broad implications for translational SCI research. Previous work has demonstrated both the univariate and multivariate impact of graded cervical SCI^13^ in rats and primates^14^,^16^, as well as a univariate assessment of a combined SCI and TBI preclinical model in rats^15^. The results of prior studies show that information about impairment of function and tissue pathology can be understood at the univariate level, and to a greater degree at the multivariate level, providing powerful opportunities for therapeutic discovery that harnesses CNS trauma big-data in ensemble. However, progress is hampered by the intensive data pre-processing typically required before and after analysis to generate a full view of the multidimensional syndromic state in CNS injury^37^. The difficulty of sophisticated analytics may partially account for the slow progress to both understand and successfully treat these complicated CNS injury syndromes. Typical CNS injury studies generate enormous quantities of data, yet only a few of these measures are assessed at a time. The ability to interpret the full pattern of disease pathology and recovery is further confounded by basic visualization attempts using bar graphs and/or recovery curves combined with potentially inappropriate statistical techniques to detect significant effects^38^ in a manner that is at once both prone to false-positives (familywise type-1 error) and wasteful of information (multivariate type-2 error)^39^. Taken together, these factors may hinder progress to rapidly translate promising preclinical studies into clinical trials, and may point researchers in the wrong direction regarding the conclusions that can be drawn from their studies.

TDA applies the mathematical concepts from geometric topology to unlock relationships in data that would be considered as noise by traditional parametric approaches such as regression and GLMs^40^. By extracting the fundamental shape from the entire multidimensional data set, TDA ascribes meaning to an otherwise unforeseen pattern of relationships among individuals. The TDA algorithm achieves this goal by iterating through multiple views of lower dimensional shape of the data to extract the persistent shape of the syndromic space across these multiple views using ensemble machine learning. Through this process, TDA can resolve meaningful signal from ‘noise' by identifying its true source, improving our understanding of the whole data set. TDA has been used previously to navigate complicated, high-dimensional biological data sets including functional brain connectivity^41^,^42^ and biomolecular folding pathways^43^. Novel applications for TDA in precision medicine are also beginning to appear in the literature. For example, TDA has been used to uncover novel relationships in immune cell reactivity between patients with type-1 and type-2 diabetes^44^, and in identifying novel subgroups of patients with asthma and the unique relationships of specific T-cell mediated interleukins with these patient subgroups^45^. Another prominent example of TDA's application towards precision medicine was found in breast cancer data regarding genetic influences on patient survival that had not been previously identified, even-though the data sets containing this information had been publically available for over 10 years^46^. Similar methods can now be applied for neurotrauma data sets at both the preclinical and clinical level, given the emergence of large-scale multicentre repositories targeting precision medicine for the CNS^11^,^37^.

In the present paper, we expand the concepts of precision medicine to the application of TDA for preclinical translational discovery, using CNS injury data sets containing diverse information from multiple preclinical treatment trials with histopathological, functional and health outcomes. TDA allows for both rapid analysis and rapid visualization of all measures collected in a particular study to increase efficiency of recovery testing following injury, and allows drill down into subpopulation clusters for targeted hypothesis testing regarding treatment efficacy across the complex variability that exists in SCI. Due to the high dimensionality of many SCI and TBI data sets, it can be difficult to interpret which measures are sensitive to improved recovery in therapeutic trials, and whether particular subgroups are selectively responsive. As shown in the present paper, the network generated from TDA can then be harnessed to test the generality of therapies—that is, whether treatments are effective within the full syndromic space—as well as specific therapeutic features such as determining whether particular outcome measures are more sensitive to therapeutic targeting.

It should be noted that the current work does have limitations. Perhaps the most translationally significant finding was the identification of a detrimental relationship between perioperative hypertension and long-term locomotor recovery following SCI. It is unclear from mining the animal hospital records what specific mechanisms may lead to animals having hypertensive episodes during SCI operation and recovery. One potential confounder is that variability in anesthesia may impact blood pressure. Although it is difficult to completely discount this possibility in a retrospective study, there is no evidence of systematic variability in anesthesia reflected in the detailed perioperative animal care records. In addition, the MASCIS pilot study tested multiple anesthetics and developed a rigid protocol of pentobarbital anesthesia, with the contusion injury delivered at a standardized time point of 1 h, with surgical plane confirmed by areflexia for the multicentre study data presented here. However, further experimental studies are needed to assess the impact of anesthetics as a potential mediator of the perioperative hypertension–locomotion relationship^47^. Regarding the potential for TDA as a precision medicine tool applied to SCI and TBI research, the current study was performed in inbred animals with consistent graded injuries living in optimal conditions that were tightly controlled within a given study (but highly variable across centres and strain). This intrinsic multicentre variability is useful for providing a proof-of-concept validation of TDA for neurotrauma in the face of potential cross-laboratory variance. However, it remains an open question whether TDA could overcome high variability seen in human clinical data for SCI and TBI, though previous studies using TDA in other diseases demonstrate its value for clinical decision support^44^,^45^,^46^.

In conclusion, rapid visualization and analysis of CNS injury big-data may facilitate rapid, accurate big-data analysis of preclinical and clinical studies, allowing for quicker validation of hypotheses tested. By exploring a large preclinical data set with multiple injury models, outcome measures and study designs, TDA discovered unique features of TBI+SCI determined by injury location, and detrimental influences of perioperative hypertension on locomotor recovery and bladder function that were previously unpublished. In this sense, TDA presents a powerful and novel bioinformatics tool for the field of neurotrauma research for testing large, heterogeneous data sets. By mapping all data collected across an entire test subject population as a multidimensional topology, TDA helps extract new knowledge about neurotrauma populations and their associated states of disease and recovery. This may expedite the translational pipeline for therapeutic discovery in neurological disease research.

## Methods

### Proof-of-concept application of TDA to neurotrauma data sets

TDA was used to rapidly analyse and visualize clustering of individuals based on their similarity across hundreds of variables simultaneously (Fig. 1). TDA is an adaptation of the methods of topology, the mathematical discipline which studies robust methods of measuring and representing shape, to create compact visual representations of high-dimensional data sets^40^,^48^. This is performed automatically within the software, by deploying an ensemble machine learning algorithm that iterates through overlapping subject bins of different sizes that resample the metric space (with replacement), thereby using a combination of the metric location and similarity of subjects in the network topology. After performing millions of iterations, the algorithm returns the most stable, consensus vote for the resulting ‘golden network' (Reeb graph), representing the multidimensional data shape^12^,^40^. The application of this method to our data sets creates clusters of subjects which appear as nodes (points) and relations among clusters are represented as interconnections (‘edges' or lines) between the nodes (Fig. 1d). Once the topological network is developed, rapid exploration of the full neurotrauma syndrome and its various manifestations across different measures can be performed (Supplementary Software 1–4). Although the application of a licensed version of TDA software was used for the present study through the Ayasdi cloud-based platform (www.ayasdi.com, v 2.0), open source versions of the program code are available in either Python^48^ or R^49^,^50^.

### TDA applied to combined TBI and cervical SCI in rats

We applied TDA to a data set containing several controlled models for combined TBI and SCI in 2–3-month-old female Long Evans rats (*n*=49, *P*=94) from a previously published study^15^. Data were analysed using the variance-normalized Euclidean metric (VNE), which finds the mean and s.d., and rescales the value of the coordinate around its mean by dividing by the s.d. of the set of values taken by the coordinate. This metric calculates the distance between two points, taking into account that each column in the data set could have significantly different variance. VNE distance between two points *X* and *Y* is given by:


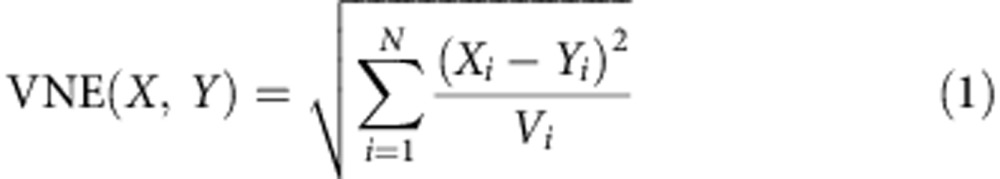


Where *V*_*i*_ is the variance associated with each column *i* and is given by:


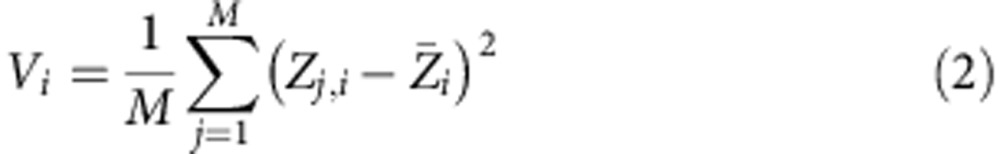


And 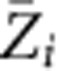 is the mean of column *i* and is given by:


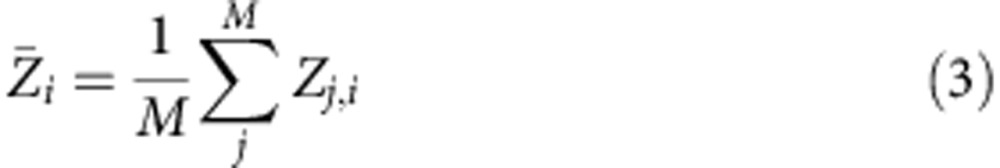


VNE was combined with the principal and secondary metric SVD lenses, which are analogous to PCA. The network was set at a resolution of 30 and a gain of × 4.0 (equalized) from which subjects with shared syndromic features were clustered together and distributed into a syndromic network topology (Fig. 2). Adjusting the resolution and gain alters the number of bins and the degree of overlap of these bins. Once the network is extracted, resolution and gain are used to ‘focus' the network similar to focusing a microscope on an image. We begin with a standard resolution of 30 and gain of 4.0 and then adjust these parameters to ensure that the majority of subjects are included in a connected node (as opposed to isolated from the network), and that all nodes are connected as a single network (if possible). Changing the resolution and gain alters the number of bins and the degree of overlap of these bins respectively, spreading subjects out across more nodes (high resolution) or forcing more subjects into each node (high gain). Network extraction, ‘focusing' and face validation of the syndromic space is based solely on primary outcomes of interest (for example, locomotion), while remaining blind to experimental conditions/predictors. In this sense we begin with the full outcome pattern and then reverse engineer the largest predictors in a data-driven manner.

Variables that were analysed included all available endpoint data, excluding predictor data such as categorical injury condition, gender or treatment. For networks in Figs 1, 2, 3, these endpoint data included injury biomechanics of brain and spinal cord tissue displacement, force and velocity, terminal tissue sparing, weight change, and 6-week time-course data points for measures of grooming, paw preference in the cylinder^13^,^51^, the Basso Beattie Bresnahan (BBB) hindlimb locomotor scale^13^,^17^, the Martinez scale of forelimb locomotion^52^ and the Irvine Beattie's Bresnahan (IBB) scale for object manipulation^53^,^54^. The majority of these variables are conceptualized and listed in Fig. 2a,c, and in the drop-down menu in the living figure Supplementary Software 1. Topologies were colour coded for each injury group,PC1 and PC2 distributions, histopathology and a few key examples of averages over time of functional outcomes (grooming, paw preference, object manipulation, forelimb and hindlimb open field). These were exported from the cloud into an HTML viewer to rapidly visualize and interpret the relationship of functional recovery to injury group and tissue pathology (Supplementary Software 1). For visualized distribution of injury models (Fig. 2c), red nodes indicated a pure population for each particular category, which included uninjured sham controls (*n*=9), mild TBI (*n*=10), unilateral 75 kdyn force-driven contusions (*n*=10), mild TBI contralateral to 75 kdyn force-driven contusion (*n*=10, SCI+TBI Contra) and mild TBI ipsilateral to 75 kdyn force-driven contusion (*n*=10, SCI+TBI Ipsi). Schematic diagrams of each injury model illustrate the placement of each injury (black ellipses) or sham controls (open ellipses) to demonstrate the laterality of each injury model that was tested.

Schematic diagrams for measures of functional recovery (Fig. 2a) and histopathology (Fig. 2b) were created for animal model visualization. Terminal outcomes were then visualized at the univariate level (Fig. 2a,b), which is the current standard in the SCI preclinical literature, showing the distribution of subjects for each injury group (Fig. 2c) for grooming, preference for the uninjured forepaw during vertical exploration in a Plexiglas cylinder, and forelimb and hindlimb locomotion in the open field (Fig. 2a). Histological measures of tissue in the brain and spinal cord, and MN sparing along the rostro-caudal extent of the injury were also plotted in the same manner (Fig. 2b).

### TDA applied to graded unilateral cervical SCI in rats

We applied TDA to graded unilateral cervical SCI in 2–3-month-old female Long Evans rats (*n*=132 subjects, *P*=119 variables, Fig. 3; Supplementary Software 2) from previously published studies^13^,^51^. Data were analysed using the norm correlation metric equation—equation (4). This metric normalizes the columns to become comparable. This metric is used when the data columns have ranges and means that vary significantly. The norm correlation (Corr) distance between two points is given by the Pearson correlation and is given by Corr (*X*, *Y*)=1−r(*X*′, *Y*′), where *X*′, *Y*′ are the column-wise, mean-centred and variance-normalized versions of *X* and *Y*.


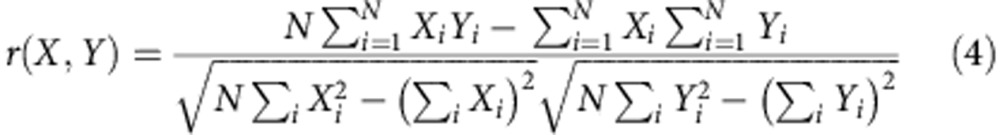


This was combined with the principal and secondary metric SVD lenses. These lenses generate a factorization of the data matrix into linearly uncorrelated components. The principal SVD lens is the highest variance component and the secondary SVD is the second highest variance component. These lenses assume that your data is using the Euclidean metric.


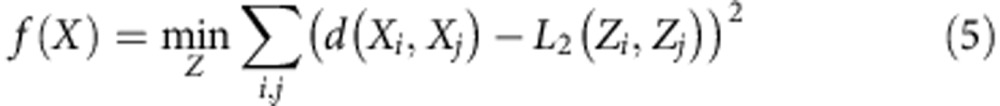


The analysis was set at a resolution of 50 and a gain of 5.0 × (equalized) from which subjects with shared syndromic features were clustered together and distributed into the syndromic network topology (Fig. 3; Supplementary Software 2, dropdown).

Variables that were analysed included all endpoint data, excluding predictor information about categorical injury condition, gender or treatment. Endpoint data used for Figs 3, 4, 5 include a standardized measure of tissue compression for injury biomechanics across different contusion devices, terminal tissue pathology measured by lesion size and white/grey matter and MN sparing, and 6-week time-course data points for measures of daily or weekly weight change, CatWalk^55^, grooming, paw preference in the cylinder^13^,^51^, BBB hindlimb locomotion^17^,^56^, a 4-point measure of forelimb locomotion^13^ and the IBB scale for object manipulation^53^,^54^. Topologies were colour coded for each injury model, PC1 and PC2 distributions, histopathology and a few key examples of functional outcomes (grooming, paw preference, forelimb and hindlimb open field) at 7, 21 and 42 DPL. These were exported from the cloud into an HTML viewer to monitor recovery of each outcome over time in relation to injury model and tissue pathology (Supplementary Software 2, dropdown). Heat maps for the colour schemes of the flares represent the range of highest values (red) to lowest values (blue) for each respective outcome being visualized (for example, lesion size; blue=0%, red=100% lesion, Fig. 3b). For visualized distribution of injury models, red nodes indicated a pure population for each particular category of graded SCI, which included uninjured sham controls (*n*=16), hemisections (*n*=9), 75 kdyn (*n*=31) and 100 kdyn (*n*=34) unilateral contusions with the force-driven impactor, and 6.25 mm (*n*=10) and 12.5 mm (*n*=32) unilateral contusions with the weight-drop impactor (Fig. 3; Supplementary Software 2, dropdown).

A detailed interpretation of the syndromic space for graded unilateral cervical SCI has been reported previously^13^, however, those analyses were performed in SPSS v. 19, and do not allow for rapid analysis and visualization of the syndromic SCI space that is presented here.

### Data-driven exploration of preclinical drug trial efficacy

Comparison of continuous variables was performed by two tests: KS test and *t*-test. The KS test was used to investigate the non-parametric probabilistic distributions of samples across each (one-dimensional) variable, while the *t*-test explores whether the null hypothesis (mean value of both samples) is supported. Comparison of categorical variables was performed by Fisher exact test. These methods were used to identify group differences from the graded cervical SCI data set between selected nodes that were classified based on a combination of purity for both injury condition (for example, 12 mm weight-drop) and treatment condition (for example, MP, minocycline, No drug; Fig. 3d, red nodes). Nodes satisfying both these criteria were designated as groups and analysed for measures that differentiated the groups from each other. Significant differences between these groups were based on KS scores with the largest absolute values (0.75–1.0) and KS *P* values.

### Data-driven exploration of MASCIS as a cross-validation test

Data mined from the VISION-SCI repository^14^ for previous trials of MP in SCI resulted in identification of the MASCIS preclinical trial from the OSU testing site. This was an NIH-sponsored multicentre trial (1994–1997) to validate the contusion model for SCI using the weight-drop contusion device^19^, and to test the efficacy of pharmacological treatments for SCI. Only subjects from year 3 (1996, *N*=72) had un-blinded treatment codes in the current version of the database. A norm correlation metric and L-infinity Centrality lens (resolution 30, Gain 4.0 × , equalized) was used to generate the network from 2–3-month-old rats receiving graded thoracic (T9) bilateral contusions (12.5 and 25 mm injuries, in both males and females, across 6 MP combination treatment conditions) with 49 separate outcome measures. Only endpoint data were used in the analysis, excluding predictor information about categorical injury condition, gender or treatment. Endpoint data used in the analysis included tissue deformation injury biomechanics and vitals measured during the SCI operation, including body temperature, heart rate and blood pressure (systolic, diastolic, mean). Vitals, along with blood gases, were measured using an intra-arterial tail catheter, and averages and maximum values were taken from 15 min before injury (PreOP), at the time of injury and 15 min post injury (PostOP). Post injury functional outcomes included averages for recovery of bladder function, urine content, weight gain and locomotor recovery on the BBB scale during the 6-week time period prior to sacrifice and terminal total tissue sparing. The full list of these variables is provided in Fig. 6b and the drop-down menu of Supplementary Software 4. These data were analysed using L-infinity centrality, which groups subjects into nodes in the network using the maximal distance of each subject from all other subjects.


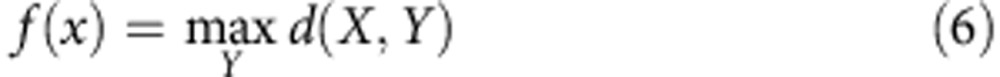


Only subjects in the vehicle (*N*=10) and MP (*N*=12) treated groups had complete data for BBB locomotion and tissue sparing for hypothesis testing about treatment effects (Fig. 4).

### Testing perioperative hypertension-recovery association

KS tests were used to compare group differences in the networks generated for the MASCIS OSU trial year 3 data set (*N*=72) to identify significant group differences between BBB functional recovery that were predicted by MAP levels at the time of injury (Fig. 5a). Rats with an age range of 2–5 months from years 1–2 of the MASCIS trial (1994–1995, *N*=154) with the same 49 outcome measures were analysed using the same TDA parameters as the 1996 data set to validate the hypothesis that elevated MAP during SCI surgery significantly predicted poorer functional recovery (Fig. 5b). Confirmation of perioperative MAP levels predicting poorer neurological recovery was performed in SPSS v. 19 using a GLM repeated measures ANOVA. The dependent variable was BBB locomotor score, time points of 1–6 weeks post injury were the repeated measures, and MAP values at either PreOP, PostOP, or at the time of injury were each used separately as covariates within the GLM and tested on each data set separately (1996 and 1994–1995). The bivariate correlation matrix comparing all variables measured over time in the entire MASCIS OSU trial (*N*=334) was generated in SPSS v.19, and two versions were overlaid to depict both Pearson correlation values and valence (Fig. 6a, red–blue heat map for positive or negative correlations, respectively), and the significance of each correlation (outlined boxes). Comparison of the bivariate correlation matrix to TDA on the same data set and set of variables measured over 6 weeks post SCI (*N*=334, *P*=150; TDA metric=norm correlation, L-infinity centrality lens, resolution 50, Gain 4.0 × , equalized) was plotted together to assess the greater efficacy of TDA to perform visually guided comparisons of the networked interactions between all test subjects based on correlations of outcome variables for a more comprehensive, holistic view and exploration of the SCI syndrome (Fig. 6b, Supplementary Software 4, dropdown).

### Statistical analysis

Statistical analysis testing between groups for the identified measures were performed in Ayasdi v2.0 for group differences in the network, and plotted for box plots or histograms in GraphPad Prism 5 and analysed for significance using two-tailed *t*-tests and one-way ANOVAs in SPSS v19 (Figs 3, 4, 5).

## Additional information

**How to cite this article:** Nielson, J. L. *et al*. Topological data analysis for discovery in preclinical spinal cord injury and traumatic brain injury. *Nat. Commun.* 6:8581 doi: 10.1038/ncomms9581 (2015).

## Supplementary Material

Supplementary Software 1 TBI-SCI syndromic network topology. An HTML viewer exported from the TDA to visualize functional recovery and histopathology of a combined unilateral mild TBI and unilateral 75kdyn force-driven SCI contusion preclinical model in rats. Results for the deficits caused by this model have been reported previously (Inoue et al 2013). Here we show the novel application of syndromic TDA to visually characterize the network topology of this emerging preclinical model to confirm the published findings. The drop-down menu (upper-right) allows for rapid exploration through all outcomes and injury models within the network. Combined SCI+TBI ipsilateral to each other restores balance of function between the forelimbs on measures of skilled forelimb function, including paw preference, grooming and object manipulation.

Supplementary Software 2 Cervical SCI syndromic network topology. The HTML viewer was exported from the TDA, which contains a drop-down menu (upper right) that allows for rapid exploration through all outcomes and injury models within the network. This visual mapping allows for the reader to orient themselves to the location of each injury models within the topology, and scroll through each outcome over time to view the relationship to recovery. Data-driven discovery of functional deficits in forelimb openfield and grooming tasks in subjects that received 12.5mm weight-drop contusions revealed decreased MN sparing and tissue area at the lesion epicenter in subjects that received either Minocycline or Methylprednisolone, compared to no drug controls.

Supplementary Software 3 MP treatment trials in thoracic SCI SVD network. The drop-down menu (upper right) allows for rapid exploration through all outcomes and injury models within the network. In a cross-validation test the detrimental effects of methylprednisolone (MP1 and MCP) we saw no significant impact of drug on either functional recovery on the BBB (p=0.24) or tissue sparing (p=0.20) relative to vehicle control.

Supplementary Software 4 The entire thoracic SCI syndromic network topology. The drop-down menu (upper right) allows for rapid exploration through all outcomes within the network. Data-driven identification of elevated perioperative blood pressure predicting functional deficits following thoracic contusions was uncovered in 2 separate datasets (Figure 5). The relationship between blood pressure and functional deficits was confirmed when all datasets were combined, and assessed over time (Figure 6). All outcomes for tissue sparing, locomotor recovery, bladder function and perioperative vitals and blood gases were mapped onto the network topology for rapid exploration of the relationship between autonomic complications and recovery of locomotion (N=334).
