# Supplementary material for: Topological data analysis for discovery in preclinical spinal cord injury and traumatic brain injury
Source: Nat Commun. 2015 Oct 14;6:8581. doi: 10.1038/ncomms9581 (PMC4634208; doi:10.1038/ncomms9581)
Supplement: Supplementary Software 1 — TBI-SCI syndromic network topology. An HTML viewer exported from the TDA to visualize functional recovery and histopathology of a combined unilateral mild TBI and unilateral 75kdyn force-driven SCI contusion preclinical model in rats. Results for the deficits caused by this model have been reported previously (Inoue et al 2013). Here we show the novel application of syndromic TDA to visually characterize the network topology of this emerging preclinical model to confirm the published findings. The drop-down menu (upper-right) allows for rapid exploration through all outcomes and injury models within the network. Combined SCI+TBI ipsilateral to each other restores balance of function between the forelimbs on measures of skilled forelimb function, including paw preference, grooming and object manipulation. [file ncomms9581-s1.zip › Supplementary Software 1.html]

Ayasdi Iris Snapshot


Groups

Notes

Pan Mode

Select Mode

Ayasdi HTML Viewer is optimized for a desktop browsing experience.  
Please use a non-mobile web browser to view.

?

Animate

ON

OFF

Change Color Scheme  
Toggle Pan or Select  
Center Network

Copyright 2013 Ayasdi Inc.

Selection Summary

0 nodes
